# A population-representative survey on attitudes toward genomic newborn screening in Germany

**DOI:** 10.1016/j.gimo.2026.104371

**Published:** 2026-01-30

**Authors:** Elena Sophia Doll, Karla Alex, Carlotta Julia Mayer, Heiko Brennenstuhl, Ulrike Mütze, Hadley Stevens Smith, Charlotte Raithel, Elmar Brähler, Ralf Müller-Terpitz, Stefan Kölker, Christian P. Schaaf, Eva C. Winkler, Beate Ditzen, Julia Mahal

**Affiliations:** 1 Heidelberg University, Heidelberg, Germany; 2 Institute for Medical Psychology, Heidelberg University Hospital, Heidelberg, Germany; 3 Heidelberg University, Faculty of Medicine, Institute for Medical and Data Ethics, Heidelberg, Germany; 4 Institute of Human Genetics, Heidelberg University, Heidelberg, Germany; 5 Heidelberg University, Medical Faculty, Center for Child and Adolescent Medicine, Division of Child Neurology and Metabolic Medicine, Heidelberg, Germany; 6 PRecisiOn Medicine Translational Research (PROMoTeR) Center, Department of Population Medicine, Harvard Medical School and Harvard Pilgrim Health Care Institute, Boston, MA; 7 Department for Medical Psychology and Medical Sociology, University of Leipzig, Leipzig, Germany; 8 Department for Psychosomatic Medicine and Psychotherapy, University Medical Center of the Johannes Gutenberg University Mainz, Mainz, Germany; 9 Law of Economic Regulation and Media, Public Law, Faculty of Law, University of Mannheim, Mannheim, Germany; 10 German Center for Mental Health (DZPG), partner site Heidelberg/Mannheim/Ulm, Heidelberg, Germany; 11 Department of Clinical Biopsychology and Psychotherapy, Institute of Psychology, University of Zurich, Zurich, Switzerland

**Keywords:** Attitudes, Genomic neonatal screening, Genomic newborn screening, Knowledge, Public

## Abstract

**Purpose:**

This study assessed general attitudes toward genomic newborn screening (gNBS) among the German public.

**Methods:**

In a population-representative survey, we assessed self-rated prior knowledge of gNBS, newborn screening (NBS), and genome sequencing and perceived attitudinal and informational uncertainty and agreement with potential advantages and disadvantages of gNBS via paper-based questionnaire. Sociodemographic information was obtained through interviews. Analyses included descriptive statistics and multiple linear regressions.

**Results:**

Respondents (*N* = 2504; ages 16-92) rated their prior knowledge as low, especially for gNBS compared with NBS. Informational uncertainty was higher among individuals with lower self-rated prior knowledge, lower educational attainment, and lower income, whereas attitudinal uncertainty showed no significant correlates with sociodemographic factors. Agreement with potential advantages of gNBS outweighed that of potential disadvantages. Higher self-rated prior knowledge and income predicted higher agreement with advantages. Right-leaning political views correlated with greater agreement with disadvantages. Overall, sociodemographic factors explained little variance.

**Conclusion:**

Perceptions of gNBS were predominantly positive, although marked by uncertainty and low prior knowledge. Although some sociodemographic differences emerged, their influence on attitudes was limited. Additional factors, such as personality traits, may help explain attitude differences. Given low prior knowledge and its association with support for gNBS, improving genetic literacy through diverse outreach strategies is essential to enhance public acceptance of gNBS in Germany.

## Introduction

Newborn screening (NBS) is one of the most successful mechanisms of secondary prevention.^1^ Incorporating genome sequencing into newborn screening (gNBS) is a rapidly advancing field of research. It is driven by technological advances and decreasing cost of next-generation sequencing technology, allowing for broad and efficient genomic analysis at large scale.^2^ As an addition to traditional, primarily biochemical NBS, it aims at identifying a wider range of genetic disorders at an early stage, potentially expanding the spectrum of conditions screened for and offering new preventive or therapeutic opportunities.^2^^,^^3^ Recent developments reflect a growing interest in integrating gNBS into public health. However, they also raise several challenges, including informed consent design, access to adequate counseling, and decisions on including untreatable or late-onset diseases.^4^, ^5^, ^6^, ^7^

These concerns have prompted debate around the gNBS implementation and highlight the need to understand public and parental attitudes toward this technology, which are explored in a growing body of research using both qualitative and quantitative methods. Qualitative studies have provided deeper insights into the perceived benefits, concerns, and preferences regarding the type of genetic information, and emotional and informational needs of parents with regard to gNBS.^8^, ^9^, ^10^, ^11^, ^12^ Quantitative studies have measured attitudes using scales that assess agreement with potential advantages and disadvantages of gNBS,^13^^,^^14^ along with motivations to pursue gNBS.^13^ Additionally, some studies have gauged more general acceptance or rejection of gNBS,^15^ whereas others have utilized semantic differential scales, asking participants to rate the screening using pairs of polarized adjectives on expanded NBS^16^ and on genome sequencing in health care in general.^17^

Previous studies report generally high levels of support, often linked to perceived clinical and familial benefits,^8^^,^^10^^,^^15^^,^^18^, ^19^, ^20^ but concerns regarding issues such as data privacy, long-term storage, impact on the family, and insufficient support after complex or uncertain findings.^8^^,^^10^^,^^21^ Similar to existing genetic diagnostic programs, concerns have also been expressed regarding potential impact of such feedback on family relationships and the pursuit of treatment options.^8^^,^^13^^,^^22^

Positive attitudes toward gNBS in mothers were associated with higher education, younger age, greater genetic knowledge**,** prior experience with genetic testing, and having fewer children.^13^ However, sociodemographic patterns in public attitudes remain underexplored, and although qualitative research has provided valuable insights into personal values, preferences, and informational needs, there has been little population-based research into broader public sentiment and the factors that influence it. Such data would allow for comparison between societies and health systems as to public attitudes toward gNBS, potentially reflecting different moral, legal, and health economic considerations.

Building on a previous qualitative focus group study conducted in Germany exploring perspectives of parents, health care professionals, and patient representatives in depth,^8^ this study aims to examine general attitudes toward gNBS and their associations with sociodemographic variables in a population-representative sample in Germany. Attitudes were assessed using various items describing potential advantages and disadvantages of gNBS. Potential associations between attitudes and sociodemographic information could be relevant for designing targeted educational strategies on gNBS in Germany. Given the complexity of attitudes toward gNBS and limited research on associations with sociodemographic variables, we used exploratory analyses to uncover patterns that might not emerge through hypothesis-driven testing. This approach enabled us to identify potential underlying relationships that can inform future research and policy development in the field of gNBS.

## Materials and Methods

This study was part of a multitopic population survey commissioned by the University of Leipzig. The Ethics Committee of the Faculty of Medicine at Leipzig University approved the study (008/24-ek).

### Participants

A sample size of approximately 2500 participants was targeted by the client to ensure a representative sample. Because the sample was predetermined for the purpose of conducting a representative survey, no specific power calculation was performed by the investigators for this study. Sample representativeness was ensured through probability sampling using a 3-stage ADM procedure (see section data collection and analysis).^23^ The ADM (“Arbeitskreis Deutscher Markt- und Sozialforschungsinstitute”) sampling system is a reference system for representative studies in Germany. Participants were eligible if they were at least 16 years old and provided informed consent. Informed consent was obtained from all participants.

### Instruments

The survey included items to measure self-rated prior knowledge of topics related to gNBS, attitudes toward gNBS, and sociodemographic characteristics. Items assessing prior knowledge and attitudes were adapted from a previous wave of the multitopic survey addressing knowledge and attitudes toward preimplantation genetic diagnosis^24^^,^^25^ and from a US study^14^ that examined beliefs about gNBS and the predictive value of these beliefs for parental intentions to pursue gNBS. At the beginning of the survey section on gNBS there was a short introduction into gNBS and the current situation in Germany (see Supplemental Information: Introduction).

#### Self-rated prior knowledge

Three items assessed participants’ self-rated prior knowledge regarding NBS, gNBS, and genome sequencing. They were phrased as follows: “Have you heard about the following topics [NBS; gNBS; genome sequencing] before today? Please estimate your level of knowledge on these topics before today.” Participants were asked to rate their prior knowledge on a scale from 0 (“I have never heard of it”) to 3 (“I was fully aware of its meaning”).

#### Attitudes regarding gNBS

In addition, 9 items were used to evaluate participants’ attitudes toward gNBS, specifically their agreement with a series of potential advantages (eg, “The introduction of gNBS could provide useful information about family health.”) and disadvantages (eg, “The introduction of gNBS would make me uncomfortable because medical personnel would examine children’s genomic data.”). For a complete list of items see results section Table 2. The items were rated on a Likert scale, ranging from 1 (“disagree”) to 5 (“agree”), with an additional option of 6 (“don’t know”). To analyze uncertainty in responses, we additionally considered response categories 3 (“partially”) and 6 (“don’t know”) separately, operationalizing attitudinal uncertainty (cat. 3, ie, lack of clear agreement or disagreement) and informational uncertainty (cat. 6). For instance, in response to the item “The introduction of gNBS could provide useful information about family health,” a selection of “partially agree” or “partially disagree” suggests attitudinal uncertainty because it reflects simultaneous endorsement and rejection of the statement. In contrast, selecting “don’t know” may indicate informational uncertainty, implying that respondents lack sufficient knowledge—about gNBS or genomic data more broadly (eg, their heritability)—to take a position. This approach captures 2 forms of uncertainty in which respondents cannot take a clear position, and both are therefore relevant for understanding public uncertainty. This approach is critically reflected in the discussion.

#### Sociodemographic items

The following sociodemographic variables were considered: age, gender, educational background, household net income, religion, place of residence (including federal state, and their classification to former West Germany/former BRD and former East Germany/former German Democratic Republic, GDR) and municipality size. We included East-West classification as a potential factor influencing attitudes toward gNBS because of the historical division and associated regime-specific socialization. Despite recent convergence, these influences have resulted in lasting differences across societal fields, including structural conditions, such as life expectancy and economic power (Federal Government Commissioner for Eastern Germany),^26^ party attachment and voter turnout,^27^ gender ideologies,^28^ and health care decision making.^29^ Additionally, political orientation was measured on a Likert scale ranging from 1 (left-wing) to 10 (right-wing).^30^

### Data collection and analysis

Data were collected via a survey conducted between March and June 2024 by the market institute Unabhängige Serviceeinrichtung für Umfragen, Methoden, und Analysen (USUMA) Berlin. The 3-step ADM design for probability sampling^23^ to ensure representativeness of the sample included (1) choosing one of 258 nonoverlapping sampling points (defined geographic areas), (2) identifying a target household within that area using a random-route procedure, (3) selecting a particular individual within that home with the Schwedenschlüssel method. The interviewer approached this individual directly to gather sociodemographic data through face-to-face interaction. After this, the interviewer provided the self-report attitude questionnaire, which the respondents completed independently while in the presence of the interviewer. The interviewer was blinded to the objectives of the study.

A weighting procedure was conducted to ensure the representativeness of the survey. This process included a transformation from household to individual level, converting the household sample into an individual sample. Weighting was then applied simultaneously based on age, gender, and federal state after a target-actual comparison with the latest micro census data. The resulting weighting variable was provided by USUMA.

All analyses were conducted in R (version 4.4.2). The weighting factor was considered in all analyses to ensure representativeness of the results, except for the sample description. Exploratory analyses were conducted, encompassing both descriptive and inferential statistics. Descriptive statistics were calculated to summarize the data and provide an overview of participants’ self-rated prior knowledge and attitudes. This involved calculating the means, standard deviations, and frequency distributions for each variable of interest. Subsequently, internal consistency (Cronbach’s alpha) was calculated for the advantages, disadvantages, and self-rated prior knowledge items separately to assess whether the respective items could be combined into a single score. Although self-rated knowledge items were rated on a 4-point scale (0-3), they were treated as quasi-interval for the calculation of mean scores, consistent with established practice in psychological and health research.^31^ As measures of uncertainty, we computed the proportion of responses in option 3 (attitudinal uncertainty) and option 6 (informational uncertainty) among all answered items. We analyzed the data using the survey package (Lumley, Gao & Schneider, 2024)^32^ to account for the survey design with sampling weights. For paired categorical comparisons of knowledge of gNBS vs. NBS, we used a design-based Rao-Scott F test, which adjusts the Pearson χ^2^ for complex survey designs. We conducted design-based paired *t* tests to compare agreement with advantages vs disadvantages, as well as uncertainty proportions between advantages vs disadvantages items. Afterward, we ran multiple regression analyses to identify predictors of self-rated prior knowledge, agreement with potential advantages and disadvantages, and attitudinal and informational uncertainty. Because of the survey structure with sampling weights, regressions were conducted with svyglm (survey package, Lumley, Gao & Schneider, 2024).^32^ To assess overall model fit, *R*^*2*^ for regression models with survey designs was calculated for each model using the fit.svyglm function (RCPA3 package; Edwards, 2024)^33^ and Wald tests were conducted, comparing the full model to an intercept-only model, via the regTermTest function (survey package; Lumley, Gao & Schneider, 2024).^32^ In all models, *P* values were adjusted via Benjamini Hochberg correction to account for multiple predictors. Only complete cases were included in the regression models. The study was pre-registered on the Open Science Framework (OSF; https://doi.org/10.17605/OSF.IO/4PF5N). R code and data set will be made available upon request.

## Results

### Sample

The study included a total of 2504 participants (1301 women, 1202 men, 1 self-reported nonbinary participant), aged between 16 and 92 years (Mean = 50, SD = 18.04). Table 1 shows further details regarding sample characteristics.

**Table 1:** Sample overview

Variable	Levels	*N*	%	Mean	SD	Value Range
Gender	Female	1301	51.96			
	Male	1202	48.00			
	Non-binary	1	00.04			
Age				49.68	18.04	16-92
	16-24 years	250	9.98			
	25-34 years	366	14.62			
	35-44 years	417	16.65			
	45-54 years	379	15.14			
	55-64 years	478	19.09			
	65-74 years	409	16.33			
	75-92 years	205	8.19			
Highest level of education	No school diploma	67	2.68			
	Lower secondary education (“Haupt/Volksschulabschluss”)	637	25.44			
	Secondary education (“Mittlere Reife/Polytechnische Oberschule”)	1037	41.41			
	Technical college degree (“Fachschulabschluss”)	109	4.35			
	Higher education entrance qualification (“Allgemeine oder fachgebundene Hoschschulreife”)	339	13.54			
	University degree	255	10.18			
	Other qualification/student/no information provided	60	2.4			
Household net income per month	Less than 750 euro	32	1.30			
	750 < 1250 euro	180	7.28			
	1250 < 2000 euro	446	18.05			
	2000 euro or more	1813	73.37			
Religion	Protestant	885	35.34			
	Catholic	687	27.44			
	Muslim	69	2.76			
	Other (eg, Jewish, Buddhist, Hindu)	51	2.04			
	No religion	804	32.11			
Nationality	German	2387	95.86			
	non-German	103	4.14			
Region: former BRD (West) and former GDR (East)	Former BRD (West)	2003	79.99			
	Former GDR (East)	501	20.01			
Type of municipality^a^	Rural community	396	15.81			
	Small town	628	25.08			
	Medium town	610	24.36			
	Large city	870	34.74			
Household with children	No children < 16 years	2014	80.43			
aged < 16 years	At least 1 child < 16 years	480	19.17			
Political orientation (left-right)^b^				5.51	1.84	1-10

a Type of municipality: rural community: 1-5000 residents, small town: 5000-20,000 residents, medium town: 20,000-100,000 residents, large city: > 100,000 residents (Federal Institute for Research on Building, Urban Affairs and Spatial Development^34^).

b Left-right political orientation:^30^ scale ranging from 1 (left) to 10 (right).

### Descriptive analysis

Table 2 shows weighted frequencies for the prior knowledge items and gNBS attitude items. A design-based Rao-Scott F test for complex survey data showed that knowledge of gNBS was rated significantly lower than knowledge of NBS, *F*(2.84, 7049.51) = 152.21, *P* <.001. Assumptions for the Rao-Scott F tests were met, with no sparsity in the weighted contingency table. Knowledge items, agreement with advantages items and agreement with disadvantages items were aggregated because of the high internal consistency within each item set (Cronbach’s α between.86 and.94). This approach allowed us to represent each construct with a single, reliable summary measure, which simplifies the regression model and helps to avoid issues of multicollinearity in regression analyses. Table 3 presents descriptive statistics for the single knowledge and attitude items and the aggregated variables (self-rated prior knowledge, agreement with advantages, agreement with disadvantages, proportions of attitudinal and informational uncertainty). The assumptions for the design-based paired *t* tests were met (complete pairs, approximately normal distribution of differences without outliers, survey design with sufficient design degrees of freedom). On average, agreement with advantages was significantly higher (Mean = 3.82, SD = 0.89) than agreement with disadvantages (Mean = 3.26, SD = 1.08), *t*(1908) = 16.08, *P* < .001, *d* = 0.39 (small to moderate effect). The proportion of attitudinal uncertainty (response option “partially”) did not differ significantly between potential advantages and disadvantages, *t*(2461) = −0.89, *p* = .376. The proportion of informative uncertainty was significantly lower for advantage than for disadvantage items (difference = −0.05), *t*(2461) = −11.11, *P* <.001, *d* = −0.24 (small effect).

**Table 2:** Weighted proportions per response options

Item	Weighted Proportions (%)			
Self-rated prior knowledge	0 (never heard of it)	1 (heard the term before)	2 (had a general idea of what it meant)	3 (was fully aware of its meaning)
NBS	56.34	27.79	13.04	4.83
gNBS	77.81	13.84	6.59	1.76
genome sequencing	76.04	14.98	6.78	2.20
Agreement and uncertainty responses per gNBS item				
Item	Agreement^a^		Uncertainty (attitudinal/informational)^b^	
The introduction of genomic newborn screening in Germany…				
(1) promotes medical and scientific progress.	47.31		20.29	24.53
(2) may allow parents to seek support and, if necessary, treatment at an early stage.	52.34		18.61	22.81
(3) can help doctors to better understand genetic diseases.	52.27		16.65	24.37
(4) can provide helpful information about family health.	48.19		18.66	24.76
(5) would help families to better plan for the future and decide on further pregnancies and family planning.	47.05		20.50	23.55
(6) would provide information about the future health of children, even if nothing is found.	44.76		22.93	26.08
(7) could lead to discrimination against children (e.g., in insurance or career choices).	30.51		18.49	30.90
(8) would make me feel uncomfortable because medical staff examine children's genome data.	27.02		20.40	27.88
(9) is contrary to the fundamental ethical values of our society.	32.32		21.67	30.00

a Response options “agree” and “rather agree.”

b Response options “partially” (attitudinal uncertainty) and “don’t know” (informational uncertainty).

**Table 3:** Weighted descriptive statistics for items and aggregated variables

Variable	M (Weighted)	SD (Weighted)	95% CI (Weighted)	Median (Weighted)	Response Range
Aggregated variables					
Self-rated prior knowledge	0.45	0.68	0.42-0.47	0	0-3
Agreement with advantages^a^	3.82	0.89	3.79-3.85	3.83	1-5
Agreement with disadvantages^a^	3.26	1.08	3.22-3.30	3.33	1-5
Proportion of uncertainty across gNBS items (%) (attitudinal/informational)^b^	0.19/0.26	0.25/0.39	0.19-0.20/0.24-0.27	0.11/0	0-1
Single items					
Self-rated prior knowledge about NBS	0.66	0.88	0.63-0.70	0	0-3
Self-rated prior knowledge about gNBS	0.32	0.68	0.30-0.35	0	0-3
Self-rated prior knowledge about genome sequencing	0.35	0.70	0.32-0.38	0	0-3
The introduction of genomic newborn screening in Germany…^a^					
(1) promotes medical and scientific progress.	3.80	1.09	3.75-3.85	4	1-5
(2) may allow parents to seek support and, if necessary, treatment at an early stage.	3.92	1.03	3.87-3.96	4	1-5
(3) can help doctors to better understand genetic diseases.	3.95	1.04	3.90-4.00	4	1-5
(4) can provide helpful information about family health.	3.82	1.09	3.77-3.97	4	1-5
(5) would help families to better plan for the future and decide on further pregnancies and family planning.	3.78	1.11	3.73-3.83	4	1-5
(6) would provide information about the future health of children, even if nothing is found.	3.56	1.12	3.51-3.61	4	1-5
(7) could lead to discrimination against children (e.g., in insurance or career choices).	3.27	1.28	3.21-3.34	3	1-5
(8) would make me feel uncomfortable because medical staff examine children's genome data.	3.09	1.28	3.03-3.15	3	1-5
(9) is contrary to the fundamental ethical values of our society.	3.38	1.20	3.32-3.44	3	1-5
(8) would make me feel uncomfortable because medical staff examine children's genome data.	3.09	1.28	3.03-3.15	3	1-5
(9) is contrary to the fundamental ethical values of our society.	3.38	1.20	3.32-3.44	3	1-5

a In calculating the mean, standard deviation, confidence interval, and median, only response values ranging from 1 to 5 were considered, while “don’t know” was excluded from these calculations.

b The aggregated uncertainty scores reflect the proportions of responses categorized as “partially” (option 3, attitudinal uncertainty) and “don’t know” (option 6, informational uncertainty) across all 9 gNBS attitude items.

### Regression analysis

Five multiple regression models were calculated for the dependent variables “self-rated prior knowledge,” “agreement with advantages,” “agreement with disadvantages,” “attitudinal uncertainty,” and “informational uncertainty.” For all models, the regression assumptions were sufficiently met. Although residual plots indicated mild deviations from homoscedasticity, these were considered acceptable because of the use of robust standard error estimations.

Table 4 shows detailed results of each model, including estimates and significance levels of predictors, and goodness of fit statistics. Higher self-rated prior knowledge was significantly associated with female gender, higher education (middle and high vs low), living in a rural community (vs larger municipalities), former West compared with former East Germany, parenthood, and more left-wing political orientation. Younger age was marginally associated with higher self-rated prior knowledge (*P* <.10). Family net income and religious affiliation were not significantly associated with self-rated prior knowledge. Higher agreement with advantages was significantly predicted by higher self-rated prior knowledge and higher family net income. There was no significant effect for gender, age, education, municipality, region, parenthood, religious affiliation, and left-right political orientation. Higher agreement with disadvantages was significantly predicted by more right- compared with left-wing political orientation. There was no significant effect for prior self-rated knowledge, gender, age, education, municipality, region, parenthood, and religious affiliation. Self-rated prior knowledge, gender, age, education, family net income, municipality, region, parenthood, religious affiliation, and left-right political orientation did not predict attitudinal uncertainty. In contrast, more informational uncertainty was significantly predicted by lower self-rated prior knowledge, lower education, and lower income. The predictors gender, age, municipality, region, parenthood, religious affiliation, or left-right political orientation were not significant.

**Table 4:** Regression models

Predictors	B	β	Std. Error	*t* value	95% CI		*p* adj.
**Model 1 (*N* = 2,354): self-rated prior knowledge**							
(Intercept)	0.28	0.24	0.11	2.59	0.06	0.50	<.01∗∗
gender (ref.: male)^a^							
Female	0.27		0.03	9.78	0.21	0.32	<.001∗∗∗
Age	−0.00	−.03	0.00	−1.75	−0.00	0.00	.095^†^
education (ref: low)^b^							
middle	0.18		0.03	6.22	0.12	0.24	<.001∗∗∗
high	0.47		0.04	10.92	0.38	0.55	<.001∗∗∗
family net income^c^	0.01	0.02	0.01	1.57	−0.00	0.03	.127
municipality (ref.: rural community)^d^							
small town	−0.11		0.04	−2.47	−0.19	−0.02	<.05∗
medium town	−0.16		0.04	−3.66	−0.24	−0.07	<.001∗∗∗
large city	−0.15		0.04	−3.67	−0.23	−0.07	<.001∗∗∗
region: former GDR (ref.: former BRD)	−0.19		0.04	−4.62	−0.26	−0.11	<.001∗∗∗
min. one child (ref.: no child)	0.26		0.04	6.19	0.17	0.35	<.001∗∗∗
no religious affiliation (ref.: religion)	0.01		0.03	0.42	−0.05	0.08	.675
left-right orientation^e^	−0.02	−0.03	0.01	−2.30	−0.03	−0.00	<.05∗
Goodness of fit: *R*^2^ = 0.15, adj. *R*^2^ = 0.15; *F*(13, 2340) = 97.60, *p* <.001							
**Model 2 (*N* = 1,922): agreement with advantages**							
(Intercept)	3.31	3.79	0.16	20.17	2.98	3.63	<.001∗∗∗
self-rated prior knowledge	0.24	.17	0.03	7.74	0.17	0.30	<.001∗∗∗
gender (ref.: male)^a^							
female	0.04		0.04	1.04	−0.04	0.13	.463
Age	0.00	.00	0.00	0.18	−0.00	0.00	.892
education (ref low)^b^							
middle (vs. low)	0.04		0.06	0.69	−0.07	0.15	.661
high (vs. low)	0.14		0.07	2.17	0.01	0.27	.106
family net income^c^	0.03	.07	0.01	3.03	0.01	0.06	<.05∗
municipality (ref.: rural community)^d^							
small town	−0.10		0.07	−1.54	−0.22	0.03	.287
medium town	−0.09		0.07	−1.45	−0.22	0.04	.357
large city	−0.04		0.06	−0.64	−0.16	0.08	.661
region: former GDR (ref.: former BRD)	−0.01		0.06	−0.14	−0.12	0.11	.891
min. one child (ref.: no child)	−0.11		0.06	−1.88	−0.22	-0.00	.171
no religious affiliation (ref.: religion)	−0.06		0.05	−1.07	−0.15	0.05	.463
left-right orientation^e^	0.01	.01	0.01	0.45	−0.02	0.03	.762
Goodness of fit: *R*^2^ = 0.06; adj. *R*^2^ = 0.05; *F*(14, 1907) = 178.25, *p* <.001							
**Model 3 (*N* = 1,827): agreement with disadvantages**							
(Intercept)	2.78	3.22	0.20	13.80	2.38	3.18	<.001∗∗∗
self-rated prior knowledge	0.07	.05	0.04	1.67	−0.01	0.14	.268
gender^a^ (ref.: male)							
female	0.11		2.09	2.09	0.00	0.22	.171
Age	0.00	.04	1.19	1.19	−0.00	0.01	.467
education (ref.: low)^b^							
middle	0.00		0.03	0.03	−0.13	0.14	.979
high	−0.04		−0.52	−0.52	−0.20	0.12	.845
family net income^c^	0.03	.01	0.25	0.25	−0.02	0.03	.923
municipality (ref.: rural community)^d^							
small town	0.10		0.18	0.18	−0.14	0.18	.923
medium town	−0.05		−0.53	−0.53	−0.21	0.12	.845
large city	−0.02		−0.20	−0.20	−0.17	0.15	.923
region: former GDR (ref.: former BRD)	0.10		1.25	1.25	−0.05	0.26	.467
min. one child (ref.: no child)	0.05		0.73	0.73	−0.08	0.20	.816
no religious affiliation (ref.: religion)	−0.13		−1.95	−1.95	−0.25	0.00	.181
left-right orientation^e^	0.05	.09	3.01	3.01	0.01	0.08	<.05∗
Goodness of fit: *R*^2^ = 0.02; adj. *R*^2^ = 0.01; *F*(14, 1812) = 60.34, *p* <.001							
**Model 4 (*N* = 2,326): attitudinal uncertainty**^f^							
(Intercept)	−1.55	−1.42	0.28	−5.47	−2.11	−1.00	<.001∗∗∗
self-rated prior knowledge	0.05	.04	0.05	1.08	−0.04	0.15	.559
gender^a^ (ref.: male)							
female	0.12		0.07	1.74	−0.01	0.26	.268
Age	−0.00	−.06	0.00	−1.67	−0.01	0.26	.269
education (ref.: low)^b^							
middle	0.18		0.09	2.02	0.00	0.36	.202
high	−0.02		0.10	−0.21	−0.23	0.19	.976
family net income^c^	0.01	.03	0.02	0.84	−0.02	0.05	.674
municipality (ref.: rural community)^d^							
small town	0.00		0.11	0.01	−0.22	0.22	.930
medium town	0.00		0.11	0.02	−0.22	0.23	.930
large city	−0.25		0.11	−2.25	−0.47	−0.03	.172
region: former GDR (ref.: former BRD)	−0.06		0.09	−0.60	−0.24	0.13	.764
min. one child (ref.: no child)	−0.07		0.09	−0.78	−0.25	0.11	.674
no religious affiliation (ref.: religion)	−0.04		0.08	−0.43	−0.20	0.12	. 845
left-right orientation^e^	0.02	.05	0.02	1.31	−0.01	0.06	.445
Goodness of fit: *R*^2^ = 0.02; adj. *R*^2^ = 0.01; *F*(14, 2311) = 12.96, *p* <.001							
**Model 5 (*N* = 2,326): informational uncertainty**^f^							
(Intercept)	0.13	−1.06	0.16	−6.59	−0.58	0.85	.718
self-rated prior knowledge	−1.31	−0.90	0.09	−10.57	−1.55	−1.06	<.001∗∗∗
gender^a^ (ref.: male)							
female	−0.10		0.10	−1.09	−0.30	0.08	.484
age	0.00	0.08	0.05	1.56	−0.00	0.01	.277
education (ref.: low)^b^							
middle	−0.39		0.12	−3.32	−0.62	−0.15	<.01∗∗
high	−0.41		0.15	−2.84	−0.70	0.12	<.05∗
family net income^c^	−0.06	−0.12	0.05	−2.48	−0.11	−0.01	<.05∗
municipality (ref.: rural community)^d^							
small town	−0.08		0.16	−0.51	−0.39	0.23	.712
medium town	0.10		0.16	0.62	−0.21	0.41	.679
large city	0.03		0.15	0.22	−0.23	0.32	.855
region: former GDR (ref.: former BRD)	0.02		0.12	0.18	−0.22	0.27	.855
min. one child (ref.: no child)	0.13		0.13	0.99	−0.13	0.40	.505
no religious affiliation (ref.: religion)	0.10		0.12	0.85	−0.13	0.33	.555
left-right orientation^e^	−0.04	−0.07	0.05	−1.37	−0.09	0.02	.344
Goodness of fit: *R*^2^ = 0.11; adj. *R*^2^ = 0.11; *F*(14, 2311) = 57.25, *p* <.001							

a Gender category “non-binary” was included in the analysis, but coefficients are not reported, as estimation was based on a single observation. Exclusion was not applied in order to prevent arbitrary omission and to reflect the diversity present in the sample.

b lower (no or lower secondary school certificate), middle (secondary school diploma), and higher education (university entrance qualification, university degree).

c 13 categories from 1 = less than 500€/month to 13 = 5000 €/month and above and treated as continuous variable for the regression models.

d Rural community: 1-5,000 residents, small town: 5,000-20,000 residents, medium town: 20,000-100,000 residents, large city: > 100,000 residents (Federal Institute for Research on Building, Urban Affairs and Spatial Development).^34^

e political orientation ranging from 1 (left) to 10 (right), ^30^ treated as continuous variable in the regression models.

f Attitudinal uncertainty reflects the proportion of “partially” (option 3) responses to the attitude items; informational uncertainty reflects the proportion of “don’t know” (option 6) responses to the attitude items.

## Discussion

In this study designed to assess general public sentiment regarding gNBS among the German population, our results show that respondents had relatively low self-rated prior knowledge of topics related to gNBS, and a substantial proportion of uncertainty was reflected in their responses. On average, across the 9 gNBS items (measuring agreement with potential advantages and disadvantages), 45% of responses reflected uncertainty, comprising 19% attitudinal (midpoint “partially” responses) and 26% informational (“don’t know” responses) uncertainty. Although both forms of uncertainty imply a lack of clear evaluative stance, we distinguished them because they might reflect different underlying components. Attitudinal uncertainty most plausibly represents ambivalence (simultaneous endorsement and rejection), whereas information uncertainty points more to puzzlement or lack of knowledge. However, we acknowledge that these modes can blur in practice, for example, some ambivalent respondents may have selected “don’t know,” and moreover, “don’t know” might also capture disinterest. This potential overlap has to be taken into consideration for the interpretation of the different correlates identified because of the separate analysis of these types of uncertainty: After *P* value adjustment, attitudinal uncertainty was not predicted by sociodemographic characteristics, whereas informational uncertainty was associated with lower subjective prior knowledge and lower socioeconomic status (ie, lower educational attainment and lower income). The implications differ accordingly: reducing informational uncertainty might require accessible, audience-appropriate information to support comprehension. Addressing attitudinal uncertainty may need different interventions, and sources of attitudinal uncertainty should be explored more in future research. Notably, parents did not exhibit higher levels of either attitudinal or informational uncertainty than respondents with no children under the age of 16 years.

Agreement with potential advantages of gNBS was generally higher than with disadvantages. The disadvantage items, however, were marked by higher informational uncertainty, whereas attitudinal uncertainty did not differ significantly between the advantages and disadvantages items. A range of sociodemographic factors were associated with prior knowledge. Indication of prior knowledge and higher income were significantly associated with agreement with advantages of gNBS, whereas right-wing political orientation correlated with higher agreement with disadvantages. Overall, the explained variance by the regression models is low. This may indicate that factors beyond sociodemographics contribute to the outcomes under study and might play a role in evaluations of gNBS

### Self-rated prior knowledge

Low levels of self-rated prior knowledge are understandable in case of gNBS and genome sequencing, given their novelty and limited public exposure. Notably, self-rated prior knowledge was also low for NBS (>50% have never heard of it), despite nearly universal uptake in Germany^35^ and few participants born before its introduction in the late 1960s. Prior knowledge was higher among parents, who are the primary decision makers in the context of NBS, compared with individuals without children under the age of 16 years. Enhancing awareness about NBS would be desirable, but more importantly, relevant information should reach expecting parents in due time. Low levels of knowledge about NBS have also been reflected in previous research in different countries.^36^, ^37^, ^38^, ^39^ Public awareness is particularly crucial in the context of gNBS because of the sensitive, predictive nature of genetic information, which can affect not only the screened individuals but also their families.

In our study, parents (with children under 16 years) rated their prior knowledge higher than nonparents. Self-rated prior knowledge was significantly associated with all assessed sociodemographic factors, except religious affiliation, income, and age. For example, women reported higher knowledge, which aligns with women are being the primary medical decision maker in families.^40^^,^^41^ It would be interesting to understand how this affects decision making within couples.

Left-leaning individuals self-reported greater prior knowledge of topics associated with gNBS, consistent with findings by Chapman et al^42^ (2019), who assessed genetic knowledge and personal engagement with genetics in individuals from 78 countries. Political orientation might affect information seeking or openness regarding novel medical technologies.

Surprisingly, participants from rural areas reported higher prior knowledge of NBS, gNBS, and genome sequencing than those from urban areas. This contrasts with previous studies indicating lower awareness or less favorable attitudes toward genetic testing among rural populations,^43^^,^^44^ although differences disappeared when adjusting for education and income in a study by DiBiase et al^45^ (2023). Possibly other factors—such as differences in health communication channels, community engagement, or personal experiences with the health care system—might explain higher self-rated knowledge in rural populations in our study. Future research should explore how health-related information is exchanged across demographic contexts and which sources are most effective to better understand communication dynamics and knowledge acquisition around topics such as NBS and gNBS. It is also important to note that, in our study, prior knowledge was self-rated rather than objectively measured. Thus, discrepancies in self-assessment accuracy may represent an alternative explanation for the observed municipal differences. To clarify this, future studies could compare subjective and factual knowledge.

Further regional differences showed lower prior knowledge in former East vs former West Germany. This may relate to prior findings of lower engagement in shared decision making in former East Germany,^29^ where a bidirectional relation is conceivable: lower prior knowledge might hinder active participation in decision making, whereas limited involvement in decision-making processes may, in turn, limit knowledge acquisition. However, findings on such regional differences regarding shared decision-making preferences are inconsistent.^46^ For future research, it would be interesting to explore actual uptake rates of gNBS, if implemented in Germany, in former East vs former West Germany.

Although religious affiliation was not significantly associated with self-rated prior knowledge in our study, Chapman et al^42^ (2019) found that genetic knowledge varied significantly across religious groups. Nonbelievers scored slightly higher than believers, although these differences accounted for only a small proportion of the overall variance, and further differences were observed between specific religious groups.

### Public responses to potential advantages and disadvantages

Potential advantages received more approval compared with potential disadvantages, informational uncertainty was significantly more frequent for disadvantages; attitudinal uncertainty, in contrast, did not differ significantly between advantages and disadvantages. This underscores the need for clear communication of risks such as discrimination and data handling, both addressed in the disadvantage items. Similarly, our previous focus group findings likewise revealed prevalent uncertainty about gNBS challenges, including data storage and untreatable conditions.^8^

In our study, higher prior knowledge was associated with higher agreement regarding potential advantages and less informational uncertainty, but not with agreement with potential disadvantages or attitudinal uncertainty. This partially aligns with previous research: in a Finnish study, higher levels of knowledge regarding genetics were associated with more nuanced attitudes, expressing both more enthusiasm and more skepticism, compared with those with lower levels of knowledge.^47^ Moreover, participants with lower levels of knowledge about genetics struggled more to form clear opinions and attitudes toward genetic testing. In our study, this pattern appeared mainly for informational uncertainty, whereas subjective prior knowledge did not predict attitudinal uncertainty. Similarly, Rose et al^48^ (2005), in a study about genetic testing for cancer risks, found that after adjustment for sociodemographic characteristics and family cancer history, higher knowledge was correlated with more positive attitudes toward genetic testing, but not with negative attitudes.

Previous research found that higher education and income were associated with more positive attitudes toward gNBS^13^^,^^49^ and genetic testing in general.^45^^,^^50^ However, the relationship may be more complex, with higher socioeconomic status being associated with more positive attitudes toward genetic testing with clear medical benefit, whereas the opposite direction may hold true for tests with no clear medical benefit.^51^ In our study, higher income was significantly associated with greater support for potential advantages of gNBS, whereas the association with education was not statistically significant after correcting for multiple testing. Nonetheless, these findings emphasize the importance of promoting understanding across all social strata by using accessible, easy-to-understand language. Future research should explore effective strategies for communicating complex genetic concepts and probability data to individuals with different levels of socioeconomic backgrounds.

Religion and political orientation may both reflect and shape underlying value systems, which might influence how individuals perceive new medical technologies. In a representative US study, high religious involvement was associated with more negative attitudes toward genetic testing.^52^ That we found no significant effect of religion may be due to the fact that our study measured only religious affiliation, not religious practice or degree of religiosity, which may be more relevant for shaping attitudes. Our finding that a more right-leaning political orientation was associated with greater agreement with disadvantages aligns with previous research showing lower interest in hypothetical gNBS^18^ and more negative views toward prenatal genetic testing^53^ among individuals with a more conservative political orientation and, more generally, with findings linking liberal political orientation to higher levels of the personality trait “openness.”^54^

### Strengths and limitations

Because of budget constraints and participant burden, especially given that the study was embedded within a larger survey, we had to strictly limit the number of items. As a result, we could not include preference measures regarding the inclusion of target diseases and emotions associated with gNBS, the number of advantages and disadvantages was unbalanced, and the introduction remained brief. Moreover, prior knowledge was assessed subjectively, which may not have accurately reflected participants’ actual level of understanding. Nevertheless, all participants received basic information, and previous research has shown positive correlations between self-rated understanding and genetic knowledge.^55^ Although potential overlap with sociodemographic variables, we included self-rated prior knowledge because of its practical relevance as a directly addressable, modifiable factor in the analyses of agreement with potential advantages and disadvantages and uncertainty. Despite these limitations, a key strength of the study is its large, population-representative sample, with all analyses accounting for sampling weights. High data quality is further supported by the fact that interviews and questionnaires were conducted during home visits, ensuring that participants could complete the survey in a focused manner. However, this setting may also introduce certain limitations. The presence of an interviewer might elicit socially desirable responses or reduce openness on sensitive issues. Moreover, conducting assessments in participants’ homes may have led to variability in environmental conditions (eg, distractions, privacy) that could have influenced responses. Nonetheless, we consider these effects to be limited, as privacy-preserving administration (ie, self-completion without direct monitoring), interviewer training, and standardized procedures aimed to minimize such biases.

### Implications and future research

Advances in the field of human genetics and decreased costs have led to wider use of genetic and genomic testing.^2^ Given that, in this study, higher levels of self-rated prior knowledge were found to be linked to greater agreement with benefits and reduced informational uncertainty, the currently limited public understanding highlights the need to enhance genetic literacy. Although decisions regarding gNBS are ultimately made within families, broader public understanding about gNBS is likely to foster individual and familial decision making. Chapman et al^42^ (2019) suggest strengthening genetic literacy through education of professionals, creating a potential cascade effect within society. This could, for example, involve gynecologists and pediatricians, who are primary points of contact for (expectant) parents, although feasibility might be bounded by capacity constraints. Establishing more forums for public engagement with genomics, including discussions about its everyday relevance, has also been recommended.^56^ Previous literature highlights the need for balanced presentation of benefits and risks of genetic testing.^57^ Given persistent informational uncertainty about disadvantages such as data handling, future gNBS outreach should clearly address risks and risk management. Especially for general information relevant to a broad patient population, educational videos, which can be produced at low cost and made easily accessible to large groups, have proven to be beneficial.^58^

Previous studies have also reported incomplete understanding of NBS.^8^^,^^36^^,^^37^ Because gNBS can detect a broader range of diseases and comes along with more complex implications than NBS, the need for education and informed consent about gNBS has been highlighted in previous research.^20^ Furthermore, in light of technological advances and the expansion of screening programs, there has been a call to promote public understanding of NBS.^36^ However, concerns have been raised that more detailed informed consent for gNBS could reduce uptake rates for NBS.^20^^,^^59^ Yet, a randomized clinical trial investigating educational interventions on NBS found higher knowledge and higher support, with no increase in refusal rates, but higher trust in the program and research use of blood spots in the experimental compared with usual care group,^60^^,^^61^ and surveying parents on genome sequencing did not reduce uptake of NBS.^62^ Based in experiences from previous research, proposed outreach strategies for NBS—such as developing online resources, leveraging social media platforms, and creating easily shareable materials such as videos and infographics to generate a multiplier effect^63^—may likewise be valuable for communication about gNBS.

Because our regression models explained only a small proportion of variance in attitudes toward gNBS, additional predictors likely play a role. Future research should examine potential additional factors, such as personality traits (eg, openness), emotions, and contact with individuals with chronic illnesses, to better understand attitudes toward gNBS. Prior focus group research^8^ discussed social influences as a contributing factor for parents’ opinion formation, suggesting that how dynamics within couples or caregiver systems may shape attitudes and behaviors toward gNBS warrant further study. Furthermore, exploring where people acquire their knowledge—and how information sources shape attitudes—could help tailor educational interventions. A more nuanced distinction between specific, self-referential attitudes and intentions, as opposed to general attitudes based on more abstract considerations about potential advantages and disadvantages, would also be valuable. A final important takeaway of this study with its focus on socio-specific factors is that, despite low levels of knowledge and general uncertainty, gNBS is predominantly perceived positively. Given the high potential of gNBS for health prevention, providing parents with an education and counseling protocol that addresses their individual concerns could prevent uncertainty and, in the long term, improve health outcomes.

## Data Availability

Data and materials are available upon request.

## Conflict of Interest

The authors declare no conflicts of interest.
