# Supplementary material for: A population-representative survey on attitudes toward genomic newborn screening in Germany
Source: Genet Med Open. 2026 Jan 30;4:104371. doi: 10.1016/j.gimo.2026.104371 (PMC12992999; doi:10.1016/j.gimo.2026.104371)
Supplement: Supplemental Material [file mmc1.docx]

**Supplementary Information 1: Introduction**

At the beginning of the survey section on genomic newborn screening (gNBS), participants were provided with the following background text: “Now we would like to ask you about a type of screening conducted after birth, known as genomic newborn screening. In Germany, every newborn is currently offered early detection testing for 19 specific diseases (conventional newborn screening). These diseases are identified through a blood sample taken from the newborn. All conditions included in the screening are serious and can significantly affect a child’s life, with symptoms typically appearing in early childhood. However, they are treatable – meaning severe symptoms can often be prevented. Genomic newborn screening could supplement the existing newborn screening. The key difference lies in the method used: genomic newborn screening involves analyzing the newborn’s genome – their complete set of genetic information – using a process called genome sequencing. This analysis can provide insights into the child’s risk of developing a potentially serious genetic disorder in the future. There is currently an ongoing discussion in Germany about whether genomic newborn screening should be introduced.”
